# Virtual Intervention for Vertebral frActures (VIVA): protocol for a feasibility study of a multicentre randomized controlled trial

**DOI:** 10.1186/s40814-025-01665-x

**Published:** 2025-07-05

**Authors:** Ada Sevinc, Alexandra Papaioannou, Suzanne N. Morin, Jennifer A. Watt, Raheem B. Kherani, Sheila Brien, Larry Funnell, Lehana Thabane, Lauren A. Beaupre, Jenna C. Gibbs, Heather Keller, Caitlin McArthur, Matteo Ponzano, Sonia Singh, Sharon Straus, Jenny Thain, Zachary J. Weston, Timothy H. Wideman, Christopher D. Witiw, Lora Giangregorio

**Affiliations:** 1 Department of Kinesiology and Health Sciences, University of Waterloo, Waterloo, ON Canada; 2 Division of Geriatric Medicine & Rheumatology and Department of Health Research Methods, Evidence, and Impact, McMaster University, Hamilton, ON Canada; 3 Department of Medicine, McGill University, Montreal, QC Canada; 4 Department of Medicine, University of Toronto, Toronto, ON Canada; 5 Division of Rheumatology, Department of Medicine, Arthritis Research Canada, University of British Columbia, Vancouver, BC Canada; 6 Osteoporosis Canada, Toronto, ON Canada; 7 Department of Health Research Methods, Evidence, and Impact, St. Joseph’s Healthcare Hamilton, McMaster University, Hamilton, ON Canada; 8 Department of Physical Therapy, Faculty of Rehabilitation Medicine, University of Alberta, Edmonton, AB Canada; 9 Department of Kinesiology and Physical Education, McGill University, Montreal, QC Canada; 10 Schlegel Research Institute for Aging and Department of Kinesiology and Health Sciences, University of Waterloo, Waterloo, ON Canada; 11 School of Physiotherapy, Dalhousie University, Halifax, NS Canada; 12 School of Health and Exercise Sciences and International Collaboration On Repair Discoveries (ICORD), Blusson Spinal Cord Centre (BSCC), The University of British Columbia, Kelowna, BC Canada; 13 Department of Family Practice, Faculty of Medicine, University of British Columbia, Vancouver, BC Canada; 14 Geriatric Medicine, Department of Medicine, Knowledge Translation Program, St. Michael’s Hospital, Li Ka Shing Knowledge Institute, University of Toronto, Toronto, ON Canada; 15 Division of Geriatric Medicine, Western University, London, ON Canada; 16 Canadian Society for Exercise Physiology, Ottawa, ON Canada; 17 Faculty of Science, Wilfrid Laurier University, Waterloo, ON Canada; 18 School of Physical and Occupational Therapy, McGill University, Montreal, QC Canada; 19 Division of Neurosurgery, Department of Surgery, University of Toronto, Toronto, ON Canada; 20 Schlegel Research Institute for Aging and Department of Kinesiology and Health Sciences, University of Waterloo, Waterloo, ON N2L 3G1 Canada

**Keywords:** Osteoporosis, Vertebral fracture, Rehabilitation, Exercise, Feasibility

## Abstract

**Background:**

Vertebral fractures due to osteoporosis cause significant pain and disability. There is guidance available on the management of osteoporotic vertebral fractures, informed by systematic reviews and a consensus process. However, few studies examine whether implementing pragmatic and patient-oriented rehabilitation interventions can improve outcomes for individuals with a vertebral fracture.

**Methods:**

The purpose of the study is to investigate the feasibility of a multicentre randomized controlled trial of an 8-week virtual rehabilitation intervention for people with an osteoporotic vertebral fracture in Ontario, British Columbia, and Quebec. The design is a multicentre randomized controlled trial with two parallel groups randomized in a 1:1 ratio, stratified by centre to immediate or delayed (10 weeks after randomization) receipt of the Virtual Intervention for Vertebral frActures (VIVA). Four centres will each recruit eight individuals (total *n* = 32) over the age of 50 who have had at least one vertebral fracture in the past 2 years confirmed by a radiology report. VIVA involves once weekly virtual rehabilitation sessions delivered over 8 weeks by an exercise professional, covering four areas: pain management, safe movement, exercise, and nutrition. Exercise professional provides resources and prescribes exercise therapy for participants to implement outside of the scheduled sessions. The primary feasibility outcomes are recruitment, retention, and adherence, and criteria for success are as follows: (a) recruiting eight people per site in 5 months, (b) 80% of participants completing the trial, and (c) 75% adherence to the virtual rehabilitation sessions. The secondary outcomes include costs relative to benefit, effects on health-related outcomes (e.g. 30-s chair stand, Brief Pain Inventory, Quality of Life Questionnaire of the European Foundation for Osteoporosis-41, SCREEN-14), and outcomes related to implementation (e.g. participant and provider experience using semi-structured interviews, fidelity, number of screened and enrolled participants by referral source, fracture verification process). Descriptive analyses (e.g. mean, count, percentage) will be performed for primary feasibility outcomes and secondary outcomes.

**Discussion:**

The results will establish the feasibility of a future trial investigating the effectiveness of the VIVA intervention in people with an osteoporotic vertebral fracture.

**Trial registration:**

The trial was registered in ClinicalTrials.gov on October 21, 2024 (NCT06650410), https://clinicaltrials.gov/study/NCT06650410 .

**Supplementary Information:**

The online version contains supplementary material available at 10.1186/s40814-025-01665-x.

## Introduction

Vertebral fracture-related hyperkyphosis can impair mobility, pulmonary function, and appetite, leading to frailty progression and falls or fractures [1–3]. The prevalence of frailty among individuals hospitalized with vertebral fragility fractures is high; one study reported a prevalence of 56% [2]. Individuals with vertebral fractures are at 2.7 times higher risk of early mortality [4, 5] and 2.5 times higher risk of experiencing back pain than those without fractures [6]. Recent systematic reviews and a consensus process informed the guidance on the management of vertebral fractures from the Royal Osteoporosis Society in the UK [7–10]. However, there are very few studies examining whether implementing rehabilitation interventions can improve outcomes in people with a vertebral fracture. Pragmatic, patient-oriented strategies to improve care for people with vertebral fractures are needed, because vertebral fractures are the most common fracture due to osteoporosis [6, 11]. People with vertebral fractures, especially those with acute fractures, disability, or advanced age [12], are under-represented in rehabilitation research. A recent network meta-analysis has shown that most evidence regarding the effectiveness of treatment options for acute pain related to vertebral fractures (< 4 weeks) is low quality [13]. Unlike joint replacement, hip fracture, or cardiac disease, rehabilitation pathways for people with vertebral fracture are scarce. Further, vertebral fractures are identified in different settings (e.g. primary care, hospital, incidental X-ray) and contexts (e.g. variable access to physical therapy, specialists) [14], making rehabilitation access and delivery a challenge.

To address the need for pragmatic, patient-oriented strategies to improve care for people with vertebral fractures [4, 10, 12, 15–17], we collaborated with people with lived experiences (PWLE) and healthcare providers to codesign the Virtual Intervention for Vertebral frActures (VIVA) [18], an intervention to support rehabilitation after vertebral fracture, with a focus on pain management, nutrition, spine safe movement, and exercise. The VIVA intervention was informed by research on patients’ and providers’ experiences with, and barriers to rehabilitation after vertebral fracture [19, 20], and has embedded behaviour change techniques such as goal setting, action planning, and feedback on behaviour. VIVA can be used for an in-person, virtual, or hybrid rehabilitation approach and is adaptable based on healthcare setting, patient preferences, or access (e.g. transportation, technology). Telerehabilitation for pain or musculoskeletal conditions is effective, feasible, and acceptable [21] and may be cost-saving, but its effectiveness and implementation for the management of vertebral fractures have not been evaluated. There is a need for patient-oriented strategies to rehabilitation in people with vertebral fractures, which will inform the proposal of a future multicentre trial of VIVA using clinical effectiveness and implementation outcomes [22].

The purpose of the current study is to establish feasibility of a future multicentre pragmatic randomized controlled trial (RCT) examining the effectiveness of VIVA. The primary research question of the VIVA feasibility trial is as follows: What is the feasibility of an effectiveness-implementation [23] RCT trial of VIVA over 8 weeks (immediate versus delayed exposure) in people with an osteoporotic vertebral fracture in Ontario, British Columbia, and Quebec? A future trial will be feasible if as follows: (a) we recruit eight (8) people per site in 5 months, (b) 80% of participants complete the trial, and (c) adherence to the virtual rehabilitation sessions is 75%. Secondary outcomes are guided by reach, effectiveness, adoption, implementation, and maintenance qualitative evaluation for systematic translation (RE-AIM QuEST) [22] framework, including effect on health outcomes (e.g. mood, quality of life, physical functioning) and implementation outcomes (e.g. exercise adherence at the end of 18-week follow-up, fidelity, the proportion recruited which are ≤ 12-week versus > 12-week post-vertebral fracture).

## Methods

### Trial design

The trial is a four-centre, pragmatic [24] (Supplementary Table 1, Supplementary Fig. 1, Additional File 1) [25], pilot hybrid type 1 effectiveness-implementation trial with two parallel groups, randomized in a 1:1 ratio and stratified by centre in variable-sized blocks (ClinicalTrials.gov Identifier: NCT06650410). Participants will be randomized to the following: (1) immediate receipt of VIVA intervention or (2) wait-list usual care control and delayed receipt of VIVA 10-week post-randomization. The randomization sequence will be created and uploaded to REDCap by the trial statistician. Randomization will take place after the baseline assessment; the exercise professional delivering the intervention (not blind to allocation) will access the randomization function in REDCap to determine the participant’s allocation and communicate it to them. Outcome assessors will be blind to group allocation. Participants will not be blind to group allocation because they will know what group they are assigned to.

### Study setting

The current trial will have four recruiting centres: two in Ontario (GERAS Centre, Hamilton, (McMaster University/Hamilton Health Sciences) and Unity Health Toronto), Arthritis Research Canada in British Columbia, and the McGill University Health Centre in Quebec. All four centres are academic sites that provide routine clinical care for individuals with vertebral fractures. The University of Waterloo will be the coordinating centre.

### PWLE and knowledge user engagement

The VIVA intervention emerged from discussions with PWLE, who voiced a need for accessible education and training after vertebral fracture. We used a survey of the Canadian Osteoporosis Patient Network to identify outcomes important to patients [26] and explored the barriers to and experiences of PWLE and healthcare providers with regard to post-vertebral fracture rehabilitation. Such work informed the co-design of the VIVA intervention, which we user-tested with nine individuals with painful vertebral fractures, who reported high acceptability and usability [18]. We have assembled a team of co-investigators, collaborators, and partner organizations, including but not limited to the authors, which includes researchers, healthcare providers, fracture liaison service team members, and PWLE to serve as the steering committee, all of whom had the opportunity to provide input on the study design, intervention, comparator, and outcomes.

The wait-list control was chosen based on input from PWLE, who indicated that participants may be more willing to participate if there was a wait-list control rather than receiving no intervention.

### Participants

Individuals will be eligible to participate if they are over the age of 50; have had at least one vertebral fracture in the past 2 years confirmed by a radiology report, and willing to participate in once-weekly virtual rehabilitation sessions for 8 weeks. To ascertain vertebral fractures, the radiology report of the potential participant must be available and must confirm the presence of one or more vertebral compression fractures based on the presence of any of the following terms: moderate, severe, or endplate fracture, grade 2 or grade 3 fracture (based on the Genant semiquantitative criteria wherein grade 2 refers to 25–40% reduction in vertebral height and grade 3 refers to > 40% reduction in vertebral height) [27], ≥ 25% loss of vertebral body height, and vertebral fracture. Individuals will be excluded if they have cauda equina syndrome or spinal cord injury; had a traumatic fracture (i.e. car accident); have an active infection; have active inflammatory arthritis with a flare-up within the past 2 years; have an inability to follow two-step commands or understand instructions and are without a caregiver to support participation; are unable to communicate in English or French (English only for the British Columbia site) and do not have someone available to translate; are participating in a similar rehabilitation programme for vertebral fractures delivered by a physical therapist, clinical exercise physiologist, or kinesiologist and includes exercise; or have any surgeries planned or health problems that might cause their health to change significantly in the next 3 months.

Each site will recruit locally, but anyone living in each respective province can participate. Recruitment will occur via healthcare providers on the research team and promotion of the study to local Fracture Liaison Services, hospitals, osteoporosis or fracture clinics, primary care, endocrinologists, geriatricians, rheumatologists, and through social media, institutional websites, and academic and knowledge user networks.

Enrolled participants will be asked to continue with usual care. We will ask all participants not to enroll in a similar rehabilitation programme that includes exercise and is supervised by a physical therapist or clinical exercise physiologist throughout the study period. Participants will be asked to self-report new exercise or rehabilitation programmes they engage in. Participants who initiate exercise or rehabilitation beyond what is required in the study after randomization will remain in the trial.

### Intervention

#### Setting and supervision

The virtual one-on-one rehabilitation sessions will take place over the Zoom platform between the participant and the exercise professional (i.e. physical therapists, clinical exercise physiologists, or kinesiologists who have experience treating people with osteoporosis). The intervention includes eight-weekly sessions, each 45 to 60 min in duration. Participants will receive a technology consultation 1 to 2 weeks prior to the baseline assessment [28].

#### Overview of the VIVA intervention

The development of VIVA was informed by *Behaviour Change Wheel, Theoretical Domains Framework* [29], and *Acceptability, Practicability, Effectiveness, Affordability, Spill-over effects, and Equity* criteria [18]. The components of the intervention were informed by qualitative studies of patient and healthcare provider experiences with recovery and rehabilitation after spine fracture [19, 20], an international consensus process on the management of vertebral fractures mapping barriers to the Behaviour Change Wheel, and selecting intervention functions with a team of healthcare providers, researchers, and patients. VIVA includes four implementation strategies [29, 30]: *education* on pain management, safe movement, exercise, and nutrition; *modeling* of and *training* on exercises, safe movement, and pain management strategies; and *enablement*, such as goal setting, action planning, and self-monitoring.

VIVA includes print and video resources and a framework for goal setting, selecting exercises, and teaching body mechanics. After the baseline assessment, participants are randomized to start the intervention in week 1 (immediately post-randomization) or in week 10 (wait-list control). An exercise professional leads one-on-one once-weekly sessions (in person, via Zoom or telephone) over 8 weeks. Sessions start with brief education on a topic (e.g. safe movement, pain management, exercise, nutrition), followed by training and modeling of exercise and safe movement strategies, then goal setting, and action planning. The exercise professional prescribes exercise therapy through resources (e.g. summaries, pictures, videos) using the Wibbi app (https://wibbi.com/) or that can be emailed or mailed. Exercises are tailored to the participant’s abilities and target balance, muscle strength, and endurance of back extensor muscles and scapular stabilizers. All participant materials and communications will be available in English at the Ontario and British Columbia sites and in both French and English at the Quebec site.

##### Structure and description of the VIVA intervention

The exercise professionals will receive two 1-h training sessions and an intervention manual. During the training, the exercise professionals will learn about the structure of each session, the comprehensive content of each topic, and guidance on exercise prescription. Before virtually meeting the participant, the exercise professional will send education resources to the participant and review answers from the demographic and medical history, nutrition risk, quality of life, and falls self-efficacy questionnaires to inform intervention tailoring. Each session includes three components: (1) Education on a chosen topic supported by education materials, (2) training on or modeling of exercises, movement strategies, or pain management tips (including learning new ones or building on/progressing existing ones); and (3) delivery of behaviour change techniques to elicit maintenance of health behaviour, such as goal setting, action planning, and problem-solving to address barriers to change. At the end of each session, the participant and exercise professional will discuss the area of focus for the next session (e.g. pain management, safe movement, exercise, nutrition) and schedule the next session. The exercise professional will deliver the prescribed exercises and any educational resources (print and video) through the Wibbi app based on discussion with the participant. Participants will use the Wibbi app for self-monitoring of adherence to prescribed exercises; they can choose to receive all materials by mail and report adherence to prescribed exercise during the rehabilitation sessions directly to the exercise professional. Nutrition education includes common concerns such as unintended weight loss, loss of appetite, constipation, and strategies to manage them (e.g. examples of nutrient- and calorie-dense foods, the recommended daily intake of protein, calcium, and vitamin D). The education is supported by one-page summaries and a video series on dietary protein intake [31]. Pain management education and training will cover pain expectations and self-management tips such as avoiding prolonged sitting, slowly building activity tolerance, and applying cold or heat and cues on movement and positioning. Safe movement education and training addresses how to modify movement to reduce pain or fracture risk (e.g. example activities that may need to be modified and how to modify). There is some overlap between the categories of safe movement and pain education because movement education can be used to manage pain, but safe movement is included separately because it is not only related to pain. Individuals with spine fractures often experience fear of future fractures and want to know how to move safely and return to their activities of daily life. The examples include training sitting erect rather than with a flexed spine, hip hinge for bending, and getting up and down from the floor; education on strategies to reduce loads on the spine, hip hinge, and getting up and down from the floor; and education on strategies to reduce loads on the spine.

The selection and progression of the exercises will be based on clinical judgment and participant preference, considering the time since fracture and whether acute fracture-related pain has diminished. The exercises will target back extensor muscles, shoulder stabilizers, balance, and upper and lower body physical functioning (Tables 1, 2, 3, 4, and 5). When the exercise programme is introduced, all participants will be prescribed at least one exercise that aims to improve endurance for back extensor muscles and perform 3–10 sets of 5–10-s holds or 2–3 sets of 10–12 repetitions with at least 2–3 repetitions in reserve (Table 1) and at least one exercise for improving shoulder mobility and pectoral girdle and perform 2–3 sets of 5–10-s holds or 2 sets of 6–10 reps with at least 2–3 reps in reserve (Table 2). For balance exercises, participants will be prescribed three exercises and perform three sets for 30 s or as many reps as possible in 30 s (Table 3). Participants < 12-week post-fracture will be prescribed at least one exercise to improve lower body physical functioning and perform 2–3 sets of ~ 6–10 repetitions, with 2–3 repetitions in reserve (Table 3). Participants will be encouraged to engage in light physical activity as tolerated and limit sedentary time, and, at ≥ 12-week post-fracture, engage in ≥ 20 min/day moderate physical activity as tolerated. Participants ≥ 12-week post-fracture will be prescribed progressive resistance training exercises, at least three upper and lower body exercises, each (Tables 4 and 5). Participants will focus on form and then progress to moderate intensity (i.e. 70–80% of estimated 1 repetition maximum (RM) or 8–12 RM).

**Table 1:** Exercises for improving back extensor endurance and spinal mobility

	**Exercise**^**1**^	**Frequency**	**Repetitions**	**Sets**	**Progression**
**Starting level**	Supine head or shoulder press into floor	Three times per week	Hold positions for 5–10 s	2	Perform three sets
	Supine thoracic extension (e.g. gentle chest lift, shoulder flexion like “arm lengthener” exercise)				
	Hip and leg extension, “pressing” into ground, bed, or firm surface or extending through the leg (e.g. leg lengthener)				
	Supine lying over rolled-up towel placed lengthways along the back to unload the spine, encourage spinal extension, and stretch pectoral and front shoulder muscles	Daily	15–20 min	2–4 times throughout the day	N/A
**Intermediate**	Head to wall in the standing position	Three times per week	Hold positions for 5–10 s	2	
	Trunk extension in prone position				
**Advanced**	Modified birddog (i.e. using a chair to provide support and increase balance, perform alternate arm flexions, then alternate leg extensions)		10–12 reps	2	Perform three sets
	Bridging in supine, with care to avoid thoracic flexion				

^1^The exercise professional will pick at least one exercise and consider location of vertebrae with fracture and whether it is appropriate to have one for thoracic extension, one for lumbar extension, or both

**Table 2:** Exercises for improving shoulder mobility and stabilizing pectoral girdle

**Exercise**^1^	**Frequency**	**Repetitions**	**Sets**	**Progression**
Supine or crook lying shoulder flexion or cross-body shoulder flexion (only with pain-free range of motion) Back-to-wall shoulder flexion	Daily	6–10 reps, > 2–3 reps in reserve	2	Increase sets, progress to sitting or standing, use harder exercise band/tubing
Shoulder external rotation or face pulls	Three times per week			
Scapula retraction (e.g. sitting position with hands behind head and elbows pointing to sides)	Three times per week	Hold positions for 5–10 s	2	Perform three sets
Wall push-ups	Three times per week	6–10 reps, > 2–3 reps in reserve	2	Increase sets, perform on kitchen counter or raised surface, try push-up plus, reduce surface height

^1^The exercise professional will pick one or more exercise and consider location of vertebra(e) with fracture and range of motion at shoulder

**Table 3:** Exercises for improving balance

	**Exercise**^**1**^	**Frequency**	**Repetitions**	**Sets**
**Anticipatory control and dynamic stability**	Heel raises and toe raises on one or both feet	Three times per week	Perform exercises for 30 s or as many reps as possible in 30 s	3
	Sit-to-stand or squat			
	Standing with foot on cone or ball, moving ball with foot, toe taps on step or pillow/foam, step-up^1^			
	Avoiding and stepping over obstacles			
	Tandem walks, walking on toes or heels			
**Functional stability limits**	Reaching or weight shifting in multiple directions			
	One leg hip hinge^2^			
**Reactive control**	Standing or moving while standing on different types of surfaces, e.g. foam and pillow			

^1^The exercise professional will choose at least one exercise per category and will include sit-to-stand or squat if building lower extremity strength is a goal

**Table 4:** Exercises for improving upper body strength that can be added after 12-week post-fracture or based on clinical judgement

	Exercise^1^	Frequency	Repetitions	Sets	Initial load	Progression
Horizontal push	Push-up (wall, counter, floor)	Three times per week	6–10 repetitions, 1–3 reps in reserve	2	N/A	Step 1: Perform three sets Step 2: Progress to standing Step 3: Use harder exercise band/tubing
	Exercise band chest press				Exercise band	
Vertical press	Shoulder press (seated or standing with band)^1^					
	Incline chest press					
	Unresisted shoulder flexion and reach in supine or standing^1^				N/A	Perform three sets
Horizontal pull	Standing row^2^				Exercise band/tubing	Step 1: Perform three sets Step 2: Use harder exercise band/tubing
	Scapular retraction, protraction					
Vertical pull	Lat pull down or band pull apart					

^1^The exercise professional will pick at least one push, one pull, and one press exercise

**Table 5:** Exercises for improving lower body strength that can be added after 12-week post-fracture or based on clinical judgement

	Exercise^1^	Frequency	Repetitions	Sets	Progression
Squat	Sit to stand, ½ squat, squat	Three times per week	6–10 repetitions, 1–3 reps in reserve	2	Increase sets, increase range of motion, change position (e.g. lying to standing), reduce reliance on support object, or add resistance
	Step up or climbing steps				
	Lunges (can use a chair for support if needed)				
Hinge	Supine bridging or hip thrust^1^				
	Hip extension or hip hinge				
Hip abduction	Hip abduction				

^1^The exercise professional will pick at least one exercise per category: squat pattern, hinge pattern, hip abduction

### Data collection and management

The data will be managed using the REDCap electronic data capturing platform (https://www.project-redcap.org/). Assessments will be conducted by research staff at each site who are blind to group allocation; participants will be invited to complete assessments virtually but can choose to complete them in person if offered by the recruiting research site. Research staff at each site will administer physical assessments virtually at the Vancouver site, in-person at the Montreal site, and both in-person and virtual at the Hamilton and Toronto site. Participants will receive a link to the questionnaires via REDCap and complete them independently before the scheduled physical assessments, with the option to respond by mail or over the phone. Participants will complete physical assessments and questionnaires at baseline, 9 weeks, and 18 weeks after enrollment (Table 6 and Supplementary Fig. 2, Additional File 1). Each assessment will take approximately 45 min to complete. The exercise professionals will not be blind to group allocation, and may collect data on participant attendance, or adverse events (e.g. falls) reported to them during exercise sessions. Participants are free to withdraw anytime from the intervention, assessment, or both.

**Table 6:** Study events and timeline

Activity		Staff member	Pre-randomization		W 1–8	W 9	W 10–17	W 18
All participants	Review eligibility criteria	Staff	X					
	Obtain consent to release medical records	Staff	X					
	Verify vertebral fracture using X-ray reports	Staff	X					
	Informed consent	Staff	X					
	Technology consultation	Staff		X				
	Demographic and medical history information	Staff blinded		X				
	Questionnaires	Staff blinded		X		X		X
	Physical assessments	Staff blinded		X		X		X
	Exit interview	Staff unblinded						X
	Adverse events, falls, fractures	Staff blinded			X			
Immediate VIVA delivery	Intervention session	EP			X			
	Practice continued behaviour change						X	
Delayed VIVA delivery	Intervention session	EP					X	
Other	Fidelity	Staff unblinded			X			
	Exit interview with exercise providers	Staff blinded						X

### Primary outcome: feasibility

The feasibility outcomes are recruitment, adherence to the rehabilitation sessions, and retention. A future trial will be feasible if as follows: (a) we recruit eight people per site in 5 months, (b) adherence to the virtual rehabilitation sessions is 75%, (c) 80% of participants complete the trial. We will report recruitment as the number of participants recruited by each site, adherence as the proportion of scheduled virtual rehabilitation sessions participants attend, and retention as the proportion of participants who complete the final assessment out of the total number of participants. The exercise professional will record attendance to sessions.

### Secondary outcomes

#### Reach

We will collect data on PROGRESS-Plus factors (Place; Race/ethnicity/culture/language; Occupation; Gender and sex; Religion, Education; Socioeconomic status; Social capital) to describe participants [32]. To understand the participant population recruited and participant preferences, we will report the numbers of individuals recruited who are ≤ 12-week versus > 12-week post-vertebral fracture and those who attend outcome assessments in-person versus virtually.

#### Potential effectiveness outcomes for the main trial

- Physical function and balance: We will use the 30-s chair stand test to assess lower extremity muscle power. We will use a series of balance tests (side-by-side stand, semi-tandem stand, tandem stand) to assess balance. We have established the feasibility of virtual assessment in pre-frail and frail older adults and have performed telephone assessments [28].
- Pain: We will use Brief Pain Inventory Short Form (BPI-SF) to assess pain. We will adapt the questionnaire for individuals with vertebral fractures by replacing the word “pain” with “back pain” in questions 3 to 9. BPI-SF measures two domains: pain severity and pain interference. Pain severity is measured using four items that assess pain at its worst, at its least, on average, and now. The mean score of the four items will be calculated to determine pain severity, ranging from 0 to 10. The pain interference will be calculated as the mean score of 10 items that measure the impact of pain on various activities, ranging from 0 to 10.
- Fear of movement: We will use Tampa Scale of Kinesiophobia (TSK) to assess the participants’ fear of movement related to the pain. TSK is a self-reported questionnaire that is comprised of 17 items. The responses are measured using a 4-point Likert scale. The total score ranges from 17 to 68 and is calculated by adding each item’s scores. The lowest score of 17 represents no or little fear of movement, whereas the higher scores indicate a greater fear of movement.
- Falls: Participants will receive a monthly falls survey to report their falls using REDCap [33]. We will report the number of people who fall and rate of falls. If a fall is reported, exercise professionals will discuss the reported fall with the participant at the beginning of the rehabilitation session to confirm the details such as nature of the fall, date, and any fall-related injuries.
- Falls self-efficacy: We will use Short Falls Self-Efficacy questionnaire to assess the participants’ fear of falling. The total score is obtained by adding the scores from each of the seven items. The cut points for degrees of concern about falling are established wherein scores 7 and 8 represent a low concern, 9 to 13 indicate a moderate concern, and 28 to 64 represent a high concern for falls.
- Quality of life: We will use the Quality of Life Questionnaire of the European Foundation for Osteoporosis-41 (QUALEFFO-41) questionnaire to measure quality of life. QUALEFFO-41 is comprised of five domains including pain (e.g. the frequency and severity of pain in the past week), physical function, social function, general health perception, and mental health function. Domain scores are generated by averaging the score for each item in the domain and expressing the score on a scale from 0 to 100. The total score is calculated by summing each item in the questionnaire and then transforming the score to a scale from 0 to 100. Higher scores indicate a greater quality of life.
- Mood: We will use Patient Health Questionnaire-8 (PHQ-8) to measure depression. The PHQ-8 includes eight items on a 4-item Likert scale. For each item, the scores of 0, 1, 2, and 3 are respectively assigned to response categories of “not at all”, “several days”, “more than half the days”, and “nearly every day”. The total score is calculated by adding up the scores of each item and ranges from 0 to 24. The cut points for mild, moderate, moderately severe, and severe depression are 5, 10, 15, and 20, respectively.
- Physical activity: We will use Physical Activity Scale for Elderly (PASE) questionnaire to measure physical activity levels. The PASE involves questions on leisure time activities, household activities, and occupational activity. The total score is calculated using an algorithm that multiplies activity weights by activity frequencies.
- Nutrition risk: We will use SCREEN-14 tool to measure nutrition risk. Lower SCREEN-14 scores indicate a greater nutrition risk. The total score is calculated by adding the total score from each response. The score ranges from 0 to 64. Individuals who receive a score less than 50 are considered nutritionally at risk. A score of 2 or less from an individual response may help understand the items that put individuals at nutrition risk.

#### Adoption

We will assess the number of screened and enrolled patients by referral source.

#### Implementation

We will assess participant and provider experiences via interviews conducted using a semi-structured interview guide and adherence to the prescribed exercise via the Wibbi app (participant preference). All participants will be invited to participate in the interviews, conducted virtually using Zoom (https://www.zoom.com). The goal of the interviews is to gather feedback on the study assessments and rehabilitation sessions, including participants’ preference for method of delivery. With consent, we will record two sessions per site, selected at random, and complete a fidelity checklist [18]. We will assess the length of time to obtain radiology reports and number of ambiguous radiology reports.

#### Maintenance (short term)

We will use adherence to the exercise sessions at the end of 18-week follow-up among the immediate group as a measure of short-term adherence.

#### Resource use

We will describe costs from a public payer perspective using data collected from participants throughout the study. At baseline and week 9, participants will complete a questionnaire to report the use of direct and indirect medical resources in the past 4 weeks and iMTA Productivity Cost Questionnaire for indirect resources. Multiplying resources collected by jurisdictional unit costs in Canadian dollars will determine the total cost. At baseline, week 9, and week 18, participants will complete EQ-5D-3L, which will be used as a measure of morbidity and potentially convert to (QALY).

#### Harms

We will ask participants to report changes in health status, falls, fractures, or any other injuries. All serious adverse events will be reported to research ethics boards and the central coordinating site in accordance with their requirements.

### Recruitment and sample size

Our sample size for the current study was derived from an estimate of the required recruitment rate for the larger trial. For the future trial, a sample size of 54 in each of the intervention and control groups would allow us to detect between-group difference of two chair stands in the 30-s chair stand test with a standard deviation (SD) of 3.7 and power of 80%, which would be similar to what we have observed in prior studies of pre-frail older adults [28, 34], and is considered a minimal clinically important difference [35]. For the larger trial, we will inflate sample size by 15% to account for attrition, resulting in a sample size of 124 participants (~ 2 individuals per month, per site, across 5 sites over 13 months). To assess feasibility of recruitment in the current study, we plan to recruit 32 participants up to 5 months across 4 sites (8 participants per site). We chose a sample size of two individuals per site per month to retain the same recruitment rate as the future trial and allowed for an additional month of recruitment to be conservative. A sample size of 32 participants will also allow us to test the evaluation cut-offs for the feasibility outcomes of retention (min = 0.5, hypothesized = 0.80, required sample size = 19) and adherence (min = 0.5, hypothesized = 0.75, required sample size = 27) with an alpha level of 0.05 and power of 80% [36]. The minimal (min) values represent the lowest acceptable outcome for study feasibility, while the hypothesized values reflect the expected and satisfactory outcomes for success.

### Analyses

All analyses (Table 7) will be performed using SAS version 9.2 (Cary, NC, USA). The reporting is guided by CONSORT, SPIRIT, SAGER, and SIITHIA guidelines [37–39]. See Table 8 for Template for Intervention Description and Replication (TIDieR) checklist and Additional File 2 for SPIRIT checklist. Participant characteristics will be reported using mean (standard deviation (SD)), median (interquartile range (IQR)), or count (percentage). For the primary outcomes, we will report recruitment, adherence, and retention using count and percentage (95% confidence interval [CI]). We will use descriptive statistics and narrative or graphical means to report reach, effectiveness, adoption, maintenance, and resource use outcomes for quantitative data using an intention-to-treat approach and use content or reflexive thematic analysis for qualitative data [40]. We will report the between-group difference in effectiveness and maintenance outcomes as mean (SD) or median (IQR), percent change, and 95% CI. Analyses of between-group differences will be exploratory, and no clinical inferences will be made. There will be no interim analyses or stopping rules.

**Table 7:** Variables, hypotheses, outcomes, and methods of analysis

Variable	Criteria for feasibility/hypothesis	Outcome measure	Methods of analysis
Primary feasibility outcomes			
Recruitment	We will recruit 32 participants, 8 per site, in 5 months	Number of participants recruited at each site	Descriptive statistics
Adherence	Adherence to the rehabilitation sessions will be 75%	The proportion of scheduled sessions participant attended	
Retention	80% of our sample will complete the trial	The proportion of participants who complete the final assessment out of the total number of participants	
Secondary clinical/implementation outcomes			
Reach	N/A	• Number of participants categorized by PROGRESS-Plus factors • Number of individuals recruited who are ≤ 12-week versus > 12-week post-vertebral fracture • Number of people who attend outcome assessments in-person versus virtually	Descriptive statistics and narrative or graphical means
Effectiveness: physical function and balance, pain, fear of movement, falls (number of falls and number of people who fall), falls efficacy, quality of life, mood, physical activity, nutrition risk	Exploratory	• 30-s chair stand test, side-by-side stand, semi-tandem stand, tandem stand • Brief Pain Inventory Short Form • Tampa Scale of Kinesiophobia • Monthly falls survey • Short Falls Self-Efficacy • QUALLEFO-41 • Patient Health Questionnaire-8 • Physical Activity Scale for Elderly • SCREEN-14	Descriptive statistics and narrative or graphical means
Harms	No difference between groups	• Self-report of adverse events	Descriptive statistics
Adoption	Exploratory	• Number of screened and enrolled participants per referral source • The length of time to obtain radiology reports and number of people with ambiguous radiology reports	Descriptive statistics and narrative or graphical means
Implementation	Understanding the experiences of individuals involved in the study and adherence to the intervention sessions to inform future trials	• Semi-structured interviews with participants and exercise professionals • Proportion of prescribed exercises adhered out of the total exercises prescribed via the Wibbi app, reporting directly to exercise professional or a log • Fidelity	Reflexive thematic analyses for qualitative data Descriptive statistics and narrative or graphical means for quantitative data
Maintenance	Exploratory	• Adherence to the exercise sessions at the end of 18-week follow-up for immediate group only	Descriptive statistics and narrative or graphical means
Resource use	Exploratory	• Cost	Descriptive statistics

**Table 8:** Template for Intervention Description and Replication (TIDieR) checklist for the VIVA feasibility study

Item category	Description
Brief name	Virtual Intervention for Vertebral frActures (VIVA): Feasibility Study for a Multicentre Trial
Why	Vertebral fractures are the most common fracture due to osteoporosis. Guidance on management suggests that a rehabilitation intervention should include pain management, nutrition, exercise, and safe movement strategies in people with a vertebral fracture; however, it is not clear if implementing the intervention may improve outcomes. Thus, there is a need to conduct a trial to determine if a future randomized controlled trial testing the effectiveness and implementation of such intervention is feasible
What: Materials	VIVA covers four areas: pain management, safe movement, exercise, and nutrition. The rehabilitation sessions led by an exercise professional tackle one of the four topics based on the need and preference of the participant. The intervention includes print and video resources and a framework for goal setting, selecting exercises, and teaching body mechanics • Participant’s handouts and videos: At the end of the sessions, each participant will receive resources based on the area covered during the session (e.g. the exercise professional provides the participant a one-pager that involves a guide to encourage adequate consumption of calcium, protein, and vitamin D) • Wibbi app: Exercise professional will deliver the resources and track adherence to the rehabilitation sessions and exercise programme using the Wibbi app • Zoom: The virtual meeting platform in which the study assessments and rehabilitation sessions will be conducted • Intervention manual: A manual provided to the exercise professionals that include detailed information on how to lead the rehabilitation sessions • Data collection manual: A manual provided to the research staff that include information on how to conduct day-to-day study procedures
What: Procedures	Exercise professional leads 1:1 once-weekly sessions (via Zoom or telephone) over 8 weeks. Sessions start with brief education on a topic (e.g. safe movement, pain management, exercise, nutrition), followed by training and modeling of exercise and safe movement strategies, then goal setting, and action planning. The exercise professional then prescribes resources
Who: Provided	Rehabilitation sessions will be delivered by an exercise professional who is a physical therapist, exercise physiologist, or kinesiologist
How	The sessions will occur via Zoom, but communication via telephone is an option if a participant cannot use Zoom, or based on participant preference. An exercise professional and participant will meet in a one-on-one format
Where	The rehabilitation sessions will occur at participant’s home with virtual support from exercise professional
When and how much	The participant and exercise professional will meet once a week for 45–60 min over 8 weeks. During each session, an exercise professional will create an action plan with the participant for the following week that outlines what, when, and how much to perform for each activity *Nutrition guidance* • Participants will implement the nutrition tips daily *Pain management strategies* • Participants will implement pain management strategies daily *Spine safe movement* • Participants will implement spine safe movements when performing daily activities *Exercise to improve strength and balance* • Depending on the time after fracture and patients’ needs, participants will perform the following categories of exercises three times a week o At least one exercise for back extensor muscles Intensity: 3–10 sets of 5–10-s holds or 2–3 sets of ~ 10–12 repetitions with at least 2–3 repetitions in reserve o One or more exercise for shoulder stabilizers Intensity: Two sets of 5–10-s holds or 2 sets of 6–10 reps, with at least 2–3 reps in reserve o Three exercises for balance Intensity: Three sets of 30 s or as many reps as possible in 30 s o Three or four exercises for lower body physical functioning Intensity: 2–3 sets of ~ 6–10 repetitions, ~ 2–3 repetitions in reserve o At least three exercises progressive resistance training Intensity: 2–3 sets of ~ 6–10 repetitions with 1–3 repetitions in reserve • Participants will be encouraged to perform light PA as tolerated and/or ≥ 20 min/day moderate physical activity as tolerated
Tailoring	The one-on-one format allows for tailoring the sessions based on different needs of each participant while considering their function and the status of the vertebral fracture. For the sessions involving exercise instruction, exercises are tailored to the participant’s abilities
Modifications	N/A
How well: Planned	With consent, we will record two sessions per site, selected at random, and complete a checklist to assess intervention fidelity. Exercise professionals will receive training and will be provided the intervention manual to ensure the rehabilitation sessions and study procedures adhere to the protocol

## Discussion

The trial will build capacity for patient-oriented approaches to rehabilitation and implementation science, and a proposal for a future multicentre trial of VIVA using clinical effectiveness and implementation outcomes, adapted to different methods of delivery (virtual, in-person, hybrid, telephone), languages (English, French), practice settings (acute care, outpatient, community), and provinces, increasing its relevance to a diversity of patients, healthcare providers, and policy makers. We will publish the results of the current trial in an academic journal and present the findings in webinars for patients and conferences for researchers and healthcare professionals. We will use the results to evolve the intervention and intervention materials for future research and implementation efforts. For example, members of our team developed a care model for vertebral fragility fractures with the Fragility Fracture Network that aligns with our intervention [41]. Future dissemination efforts may include a VIVA implementation toolkit that we can disseminate with national or international organizations focused on fracture prevention.

## Supplementary Information

Additional file 1: Fig. 1. PRECIS-2 Wheel. Supplementary Fig. 2. Visual depiction of the study events and timeline. Supplementary Table 1. PRECIS-2 Tool

Additional file 2: SPIRIT 2013 Checklist: Recommended items to address in a clinical trial protocol and related documents

## Data Availability

Not applicable.

## Abbreviations

PWLE: People with lived experiences
VIVA: Virtual Intervention for Vertebral frActures
RE-AIM QuEST: Reach, Effectiveness, Adoption, Implementation, and Maintenance Qualitative evaluation for Systematic Translation
PROGRESS: Place, Race/ethnicity/culture/language, Occupation, Gender and sex, Religion, Education Socioeconomic status; Social capital
BPI-SF: Brief Pain Inventory Short Form
TSK: Tampa Scale of Kinesiophobia
QUALEFFO-41: Quality of Life Questionnaire of the European Foundation for Osteoporosis-41
PHQ-8: Patient Health Questionnaire-8
PASE: Physical Activity Scale for Elderly
QALY: Quality-adjusted life year
SD: Standard deviation
IQR: Interquartile range
CI: Confidence interval
