# Supplementary material for: Virtual Intervention for Vertebral frActures (VIVA): protocol for a feasibility study of a multicentre randomized controlled trial
Source: Pilot Feasibility Stud. 2025 Jul 5;11:94. doi: 10.1186/s40814-025-01665-x (PMC12228205; doi:10.1186/s40814-025-01665-x)

**Supplementary Table 1.** PRECIS-2 Tool

|  | Domain | Description of the domain | Score | Rationale |
| --- | --- | --- | --- | --- |
| 1 | Eligibility | To what extent are the participants in the trial similar to those who would receive this intervention if it was part of usual care? | 5 | The eligibility criteria is designed to mimic the patients who typically would receive the rehabilitation treatment in usual care. Inclusion criteria require participants to speak English or French, or have someone available to translate, which may be more limited with respect to diversity than in a clinical setting |
| 2 | Recruitment | How much extra effort is made to recruit participants over and above what would be used in the usual care setting to engage with patients? | 2 | We will advertise the study using social media, flyers, and the academic/patient networks, indicating that extra resources will be used to recruit individuals outside of the healthcare system. However, the recruitment will also occur through referrals from physicians who routinely treat patients with vertebral fractures, specialized clinics, and emergency departments. |
| 3 | Setting | How different are the settings of the trial from the usual care setting? | 5 | The study has four centres across Canada, allowing it to be conducted in French and English and in different provinces. The intervention is designed to be applicable at different formats (virtual, in-person, hybrid). |
| 4 | Organisation | How different are the resources, provider expertise, and the organisation of care delivery in the intervention arm of the trial from those available in usual care? | 5 | The exercise professionals’ have various levels of experience working with similar populations. The intervention is designed to be delivered without any extra resources necessary than what is already available to exercise professionals. |
| 5 | Flexibility (delivery) | How different is the flexibility in how the intervention is delivered and the flexibility anticipated in usual care? | 4 | The intervention is designed to be highly flexible, allowing the exercise professional to choose a topic based on participant needs and preferences. Participants will not be excluded if they start a new exercise program during the intervention period, but will be asked to report. However, there are tight limits around when the intervention is delivered (weekly for 8 weeks) which may not be the case in a usual care setting. |
| 6 | Flexibility (adherence) | How different is the flexibility in how participants are monitored and encouraged to adhere to the intervention from the flexibility anticipated in usual care? | 5 | We do not conduct exclusion based on adherence, or address poor adherence. Participants are asked to report adherence to exercise sessions, which are also conducted in a clinical setting. |
| 7 | Follow-up | How different is the intensity of measurement and follow-up of participants in the trial from the typical follow-up in usual care? | 1 | The study includes questionnaires and assessments that may not be part of the usual care setting. The study assessments are likely to be less frequent in usual care. Also, the assessments are conducted by the research staff, not the exercise professional. |
| 8 | Primary outcome | To what extent is the trial’s primary outcome directly relevant to participants? | 1 | The primary outcome of the future trial is a patient important outcome, chosen based on involvement from patients with vertebral fractures. However, the current trial is a feasibility study, so the outcomes are not directly to participants. |
| 9 | Primary analysis | To what extent are all data included in the analysis of the primary outcome? | 5 | All participants are included in the analysis. We will use intention-to-treat protocol with multiple imputation. |

**Supplementary Figure 1**. PRECIS-2 Wheel


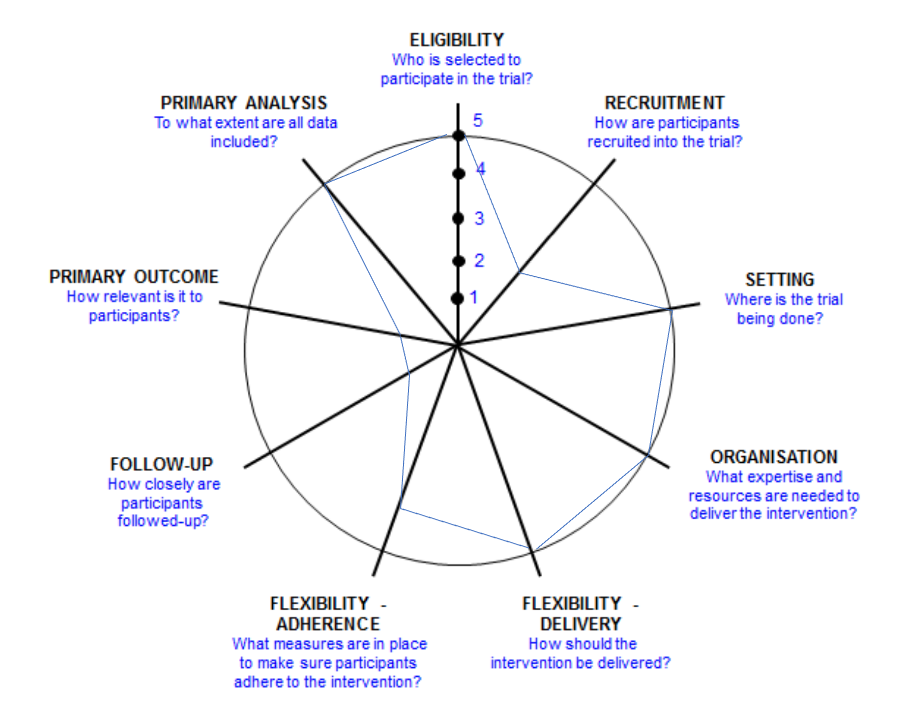


**Supplementary Figure 2.** Visual depiction of the study events and timeline


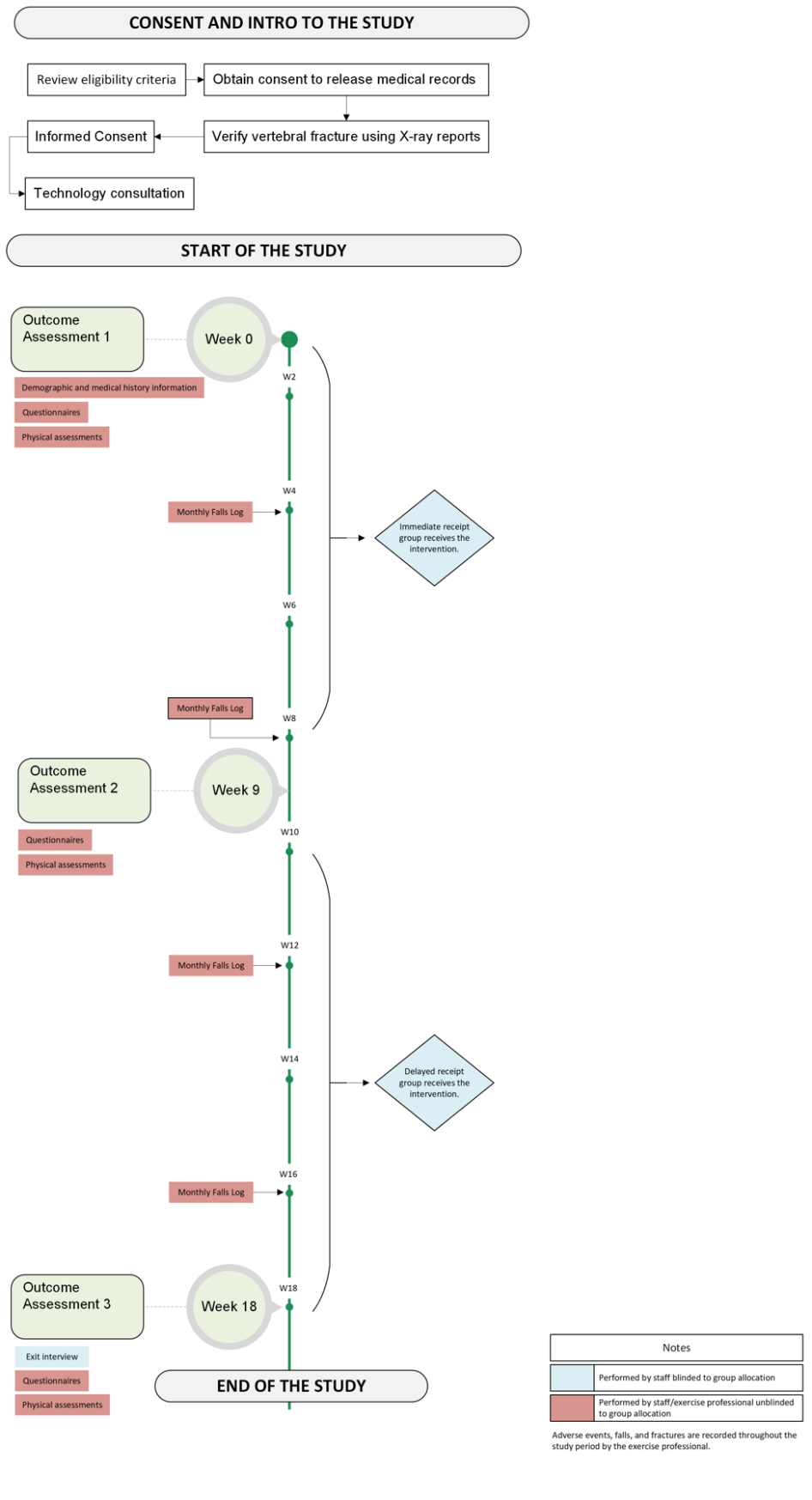

Supplement: Supplementary file 1 — Additional file 1: Fig. 1. PRECIS-2 Wheel. Supplementary Fig. 2. Visual depiction of the study events and timeline. Supplementary Table 1. PRECIS-2 Tool [file 40814_2025_1665_MOESM1_ESM.docx]
